# Albumin Fusion at the N-Terminus or C-Terminus of HM-3 Leads to Improved Pharmacokinetics and Bioactivities

**DOI:** 10.3390/biomedicines9091084

**Published:** 2021-08-25

**Authors:** Ting Li, Han-Zi Zhang, Guang-Fei Ge, Zhao-Rong Yue, Ru-Yue Wang, Qian Zhang, Yan Gu, Mei-Juan Song, Wen-Bo Li, Min-Zhi Ma, Mei-Zhu Wang, Hui Yang, Yang Li, Hong-Yu Li

**Affiliations:** 1 Gansu Key Laboratory of Biomonitoring and Bioremediation for Environmental Pollution, Institute of Microbiology, School of Life Sciences, Lanzhou University, Lanzhou 730000, China; lit2016@lzu.edu.cn (T.L.); zhanghz18@lzu.edu.cn (H.-Z.Z.); gegf17@lzu.edu.cn (G.-F.G.); wangry19@lzu.edu.cn (R.-Y.W.); qianzhang19@lzu.edu.cn (Q.Z.); songmj17@lzu.edu.cn (M.-J.S.); liwb2020@lzu.edu.cn (W.-B.L.); mamzh09@lzu.edu.cn (M.-Z.M.); 2 Gansu High Throughput Screening and Creation Center for Health Products, School of Pharmacy, Lanzhou University, Lanzhou 730000, China; yuezhr18@lzu.edu.cn (Z.-R.Y.); guy18@lzu.edu.cn (Y.G.); wangmz@lzu.edu.cn (M.-Z.W.); 3 Institute of Biology, Gansu Academy of Sciences, Lanzhou 730000, China; yanghui43@163.com

**Keywords:** fusion protein, expression, anti-tumor, half-life

## Abstract

HM-3, an integrin antagonist, exhibits anti-tumor biological responses and therefore has potential as a therapeutic polypeptide. However, the clinical applications of HM-3 are limited by its short half-life. In this study, we genetically fused human serum albumin (HSA) to the N or C-terminus of HM-3 to improve HM-3 pharmacokinetics. HM-3/HSA proteins were successfully expressed in *Pichia pastoris* and displayed improved pharmacokinetic properties and stability. Among them, the half-life of HM-3-HSA was longer than HSA-HM-3. In vitro, the IC_50_ values of HSA-HM-3 and HM-3-HSA were 0.38 ± 0.14 μM and 0.25 ± 0.08 μM in B16F10 cells, respectively. In vivo, the inhibition rates of B16F10 tumor growth were 36% (HSA-HM-3) and 56% (HM-3-HSA), respectively, indicating antitumor activity of HM-3-HSA was higher than HSA-HM-3. In conclusion, these results suggested that the HM-3/HSA fusion protein might be potential candidate HM-3 agent for treatment of melanoma and when HSA was fused at the C-terminus of HM-3, the fusion protein had a higher stability and activity.

## 1. Introduction

HM-3 (IVRRADRAAVPGGGGRGD) is a polypeptide which displayed antitumor activity in multiple animal tumor models such as melanoma and hepatocellular carcinoma [1,2]. HM-3 was constructed by the connection of RGD to the C-terminus of the ES-2 peptide with the GGGG linker. RGD is a tri-peptide sequence that has a high affinity to integrin αvβ3. ES-2, corresponding to amino acids 60–70 of endostatin, inhibits tumor cells and endothelial cells migration and invasion by binding to integrin α5β1 and αvβ3 [1,3,4]. At present, HM-3 has recently entered clinical trials in China to treat solid tumors (CTR20200847). However, the short circulating half-life of unmodified HM-3 makes frequent dosing over an extended period necessary, which place constraints on its therapeutic applicability.

Protein modification is an effective way to improve HM-3 stability in serum, such as Fc-fused HM-3 [5] and PEGylated HM-3 [6,7]. Compared with Fc and PEG, human serum albumin (HSA) is the most abundant and well-studied serum protein with a half-life around 19–22 days [8]. The long circulation persistence of HSA is enabled by its domain III interaction with the neonatal Fc receptor (FcRn) [9]. Based on this known physiological property of HSA, HSA fusion technology had been widely applied to generate long-acting therapeutic proteins and many studies indicated that the pharmacokinetic profile of small molecule drugs was significantly improved after being linked with HSA [10,11,12,13,14]. Unlike Fc and PEG, HSA is not involved in immune defense and thus is unlikely to result in an adverse immune reaction [15]. More encouragingly, two HSA fusion proteins (Tanzeum^®^ and Idelvion^®^) have been approved by the FDA for the treatment of type 2 diabetes mellitus and hemophilia B, respectively [10]. In addition, as a biological carrier, HSA is preferentially accumulated in tumor cells, indicating its potential use as a drug carrier for cancer therapy [16]. Therefore, we introduced HSA to improve pharmacokinetics of HM-3 in this study.

Fusion orientation is an important impact factor for the expression level and bioactivity of HSA fusion proteins. It has been reported that when HSA was fused at the N-terminus of TMP (thrombopoietin mimetic peptide), it resulted in a larger decline in activity than when fused at the C-terminus [17]. However, Ueda et al. [14] reported the opposite result on HSA/hLF (human lactoferrin) fusion proteins. At present, the exact reason is not clear. Thus, we chose to construct two fusion proteins (HSA-HM-3 and HM-3-HSA) to explore the effect of HSA fusion orientation on the expression and bioactivity of HM-3 fusion proteins.

The methylotrophic yeast *Pichia pastoris* is a highly successful system for the production of a variety of heterologous proteins due to their capability of protein processing mechanisms, including signal peptide cleavage, protein folding, and posttranslational modifications, within the cell. In this study, fusion proteins composed of HSA genetically fused at the C or N-terminus of HM-3 were expressed in *Pichia pastoris*. After purification, we systematically evaluated the production, properties, pharmacokinetic profiles, and antitumor bioactivity of the two fusion proteins.

## 2. Materials and Methods

### 2.1. Construction of the Expression Plasmid

DNA sequence encoding of the HSA protein was amplified using the plasmid pcDNA^TM^ 3.1-HSA as the template with primer P_HSA_ and R_HSA_. DNA sequence encoding of the HSA-HM-3 fusion protein was generated by three steps of PCR with forward primer P_1_ and reverse primer R_1_, R_2_, and R_3_. DNA sequence encoding of the HM-3-HSA fusion protein was generated by three steps of PCR with forward primer P_2_, P_3_, P_4_, and reverse primer R_4_. The sequence of primers used in this study is shown in Table S1. After that, *HSA-HM-3* or *HM-3-HSA* was cloned into the *Stu* I- and *Kpn* I-cleaved pPinkα-HC vector. The ligation mixture was transformed into TOP10 cells. The recombinant plasmid was verified by sequencing (Genewiz Co., Suzhou, China) and transformed into the *Pichiapink* strain (Thermo Fisher Scientific Co., Carlsbad, CA, USA) after linearized by *Afl* II (New England Biolabs, Ipswich, MA, USA). Recombinant clones were selected on PAD plates.

### 2.2. Expression and Purification

The *Pichiapink* expression system was used to obtain recombinant HM-3/HSA fusion proteins in soluble form. The recombinant yeast transformants were cultured in 50 mL BMGY medium at 30 °C on a shaking incubator at 300 rpm. When the OD_600_ value of the culture reached 2–6, yeast cells were harvested by centrifugation at 1500× *g* for 5 min and resuspended in 10 mL BMMY medium. The culture was continued to grow at 30 °C on a shaking incubator at 300 rpm. During this process, 2% (*v*/*v*) methanol was added to medium at a 24 h interval to supplement the carbon sources. Samples were taken periodically and the supernatant of culture was analyzed by 12% reducing SDS-PAGE, followed by Coomassie blue staining and identified by the anti-HM-3 polyclonal antibody (preparation in our laboratory) and polyclonal antibody (Sigma Co., St. Louis, MO, USA).

The supernatant containing the fusion protein was treated by dialysis against 0.02 M PB buffer (pH 7.0, 1 L 0.02 M PB buffer containing 4.37 g Na_2_HPO_4_·▪12H_2_O and 1.06 g KH_2_PO_4_). In the initial capture step, the condensed liquid was applied to an DEAE Sepharose 6 Fast Flow column (GE Healthcare Co., Uppsala, Sweden), re-equilibrated with 0.02 M PB buffer, and eluted by 0.02 M PB buffer containing 0.1 M NaCl, pH 7.0. Subsequently, the desired fraction was purified by Phenyl Sepharose 6 Fast Flow (GE Healthcare Co., Uppsala, Sweden), pre-equilibrated with 0.02 M PB buffer containing 1.5 M (NH_4_)_2_SO_4_, and eluted by 0.02 M PB buffer containing 0.1 M (NH_4_)_2_SO_4_, pH 7.0. The desired fraction was dialyzed against 0.01 M PBS (pH 7.0, 1 L 0.01 M PBS buffer containing 1.44 g Na_2_HPO_4_, 0.24 g KH_2_PO_4_, 8 g NaCl, and 0.2 g KCl) at 4 °C for 8 h. The purified samples were analyzed by 12% non-reducing SDS-PAGE by Coomassie blue staining.

### 2.3. HPLC Analysis

The purity of fusion proteins was analyzed by HPLC (Protein-Pak^TM^ 125A, 7.8 × 300 mm column, Waters Co., Milford, MA, USA). The mobile phase involved the following: 1.0 M NaCl solution; isocratic elusion, flow rate: 0.5 mL/min; temperature: 25 °C (column and detector); wavelength: 280 nm; and injection volume: 10 μL.

### 2.4. Stability of Fusion Proteins Test

The purified fusion proteins were exchanged in 0.02 M phosphate buffer with the desired pH (pH 6.0, 7.0, and 8.0) by an ultrafiltration concentration tube (Millipore, Burlington, MA, USA). The fusion proteins were adjusted to 1 mg/mL and incubated at 37 °C for different period of time (1, 5, 10, and 30 days). The structure of fusion proteins was detected by non-reducing SDS-PAGE.

### 2.5. Circular Dichroism Spectrometry

CD is based on the principle that the differential absorption of polarized light by a chiral molecule (i.e., right and left-handed rotation of circularly polarized light induced by optically active molecules in the sample) provides structural information. The secondary structure of fusion proteins suspended in PBS at a concentration of 0.1 mg/mL was assessed by circular dichroism spectroscopy (JASCO J–1500, Tokyo, Japan) using a 1 mm circular quartz cell, as described previously [18]. Scans were made from 250 to 200 nm. The applied CD parameters were as follows: bandwidth: 1 nm; scanning speed: 200 nm/min; D.I.T.: 0.5 sec; data pitch: 0.5 nm; and temperature: 25 °C (detector).

### 2.6. Cell Lines and Animals

Murine melanoma cells (B16F10) and human foreskin fibroblast cells (HFF) were purchased from American Type Culture Collection (ATCC, Manassas VA, USA). Two cells were cultured in RPMI 1640 medium modified with 10% (*v*/*v*) fetal bovine serum (FBS) and 1% (*v*/*v*) penicillin/streptomycin antibiotics and maintained at 37 °C in 5% CO_2_. Kunming mice (female, 5 to 6-weeks-old, weight 20 ± 2.0 g) were obtained from the Medical Animal Center of the Lanzhou University. C57BL/6 mice (female, 5 to 6-weeks-old, weight 18 ± 2.0 g) were obtained from Lanzhou Veterinary Research Institute, Chinese Academy of Agricultural Sciences. All animals were housed in an environmentally controlled room (24 ± 2 °C, 12 h: 12 h light/dark cycle) and clean water and standard mice chow were given every day.

### 2.7. Pharmacokinetics Analysis

Kunming mice are the most common model used to detect the pharmacokinetics of drugs. In this study, Kunming mice were randomly divided into ten group (*n* = 5) and administered with a single i.v. injection of HSA-HM-3 (0.2 μmol/kg) or HM-3-HSA (0.2 μmol/kg) in 0.1 mL of PBS. The blood samples were collected from mice eyes in intervals (2.5 min, 5 min, 30 min, 1 h, 2 h, 3 h, 6 h, 12 h, 24 h, and 48 h) after administration. The serum was obtained by the centrifugation at 4 °C, 1000 rpm, for 5 min and the supernatant was stored at −70 °C. HSA-HM-3 and HM-3-HSA concentration in serum were evaluated using the immunoenzymetric assay ELISA kit for HSA (Cygnus Co., Southport, NC, USA). Furthermore, pharmacokinetic analysis was performed with the non-compartmental modeling by DAS version 3.2.7 software. The pharmacokinetic parameter estimates derived from ELISA data included maximum concentration (C_max_), area under the time vs. concentration curve (AUC), clearance, and t_1/2_.

### 2.8. MTT Assay

The cell viability was assessed using the MTT assay. B16F10 cells or HFF cells in RPMI 1640 medium containing 10% FBS were seeded into 96-well plates (3 × 10^3^ cells/well) and cultured overnight. Then, the cells were treated with various doses of HM-3, HSA-HM-3, or HM-3-HSA and incubated for 48 h. Fusion proteins were dissolved and diluted with PBS (0.01 M, pH 7.0). Control is an equal volume of PBS. Afterwards, 15 μL 3-(4,5-dimethylthiazol-2-yl)-2,5-diphenyltetrazolium bromide solution (MTT, 5 mg/mL) was added to the 96-well plates for 4 h. The medium was discarded and 100 μL of dimethyl sulfoxide (DMSO) was added. Absorbance was read at 490 nm with the Microplate Reader (Bio-Rad). Cell viability was calculated according to the data of untreated control cells using the formula = 1 × (sample Abs)/(control). The experiment was repeated three times.

### 2.9. Colony Formation Assay

B16F10 cells in RPMI 1640 medium containing 10% FBS were seeded into the 24-well plates (200 cells/well) and incubated overnight to allow the culture to stabilize. Then, the cells were treated with HM-3 (0.25 μM), HSA-HM-3 (0.25 μM), or HM-3-HSA (0.25 μM) for 48 h. Afterwards, the medium was replaced with fresh RPMI 1640 medium supplemented with 10% FBS but without any drugs. After 7 days, the cells were washed twice with PBS. Colonies were fixed with 4% paraformaldehyde for 30 min and stained with 0.1% crystal violet for 15 min. To determine the number of colonies forming units, the clones were observed and counted. The experiment was repeated three times.

### 2.10. Cell Cycle Analysis

B16F10 cells in RPMI 1640 medium containing 10% FBS were seeded in 6-well plates (5 × 10^5^ cells/well). After 12 h, the cells were treated with HSA-HM-3 (0.25 μM) or HM-3-HSA (0.25 μM). After 48 h of incubation, the cells were harvested and fixed in 1 mL of pre-cooled 70% ethanol for 12 h at −20 °C. Then, cells were washed three times with ice-cold PBS. Afterwards, 100 μg RNAase A was added for 30 min at 37 °C and stained with propidium iodide (PI) for 30 min. Flow cytometric analysis was performed with a BD AccuriC6 flow cytometer (BD Biosciences, Franklin Lakes, NJ, USA). For each analysis, 10,000 events were collected and analyzed using Modifit LT 5.0 software. The experiment was repeated three times.

### 2.11. Migration Assay

To evaluate cell migration, wound-scratch assay was performed. B16F10 cells in RPMI 1640 medium containing 10% FBS were seeded into 96-well plates (2 × 10^4^ cells/well) and grown until 90% confluence of well was reached. Scratches were carried out by use of a white pipette tip. After wounding, the cells were rinsed with PBS and cultured with 1% FBS-containing RPMI 1640 medium supplemented with HM-3 (0.25 μM), HSA-HM-3 (0.25 μM), or HM-3-HSA (0.25 μM). After 0 h and 48 h of incubation, wound closure was monitored under an inverted phase contrast microscope. Control is an equal volume of PBS. The ratio of migration was analyzed by measuring the distance of migration in the same view at 0 h and 48 h. The experiment was repeated three times.

### 2.12. Invasion Assay

Cell invasion assay was performed using a trans-well system (8 μm pore size, Corning). B16F10 cells were trypsinized and placed at the upper chambers of trans-well insert (5 × 10^4^ cells) in 1% FBS-containing RPMI 1640 medium. HM-3 (0.25 μM), HSA-HM-3 (0.25 μM), or HM-3-HSA (0.25 μM) was added into the upper chamber. At the same time, RPMI 1640 medium containing 10% FBS was added into the lower well to function as a chemoattractant. Cells were allowed to migrate for 48 h. Non-migrated cells in the upper side were scraped with a cotton swab. The cells that migrated to the underside of the insert membrane were fixed with 4% paraformaldehyde for 30 min and stained with 0.1% crystal violet for 15 min. Cells that had migrated to the bottom of the membrane were visualized and counted. The experiment was repeated three times.

### 2.13. Integrin Antibody-Blocking Assay

The integrin antibody-blocking assay was done according to how it was described previously [19], with some modifications. Polyvinyl chloride microtiter plates (Corning, NY, USA) were coated with HM-3/HSA (400 μg/mL) overnight at 4 °C. The coated plates were washed two times with 0.9% sodium chloride and then blocked with serum-free RPMI medium containing 5% BSA for 1 h at 37 °C. After washing again, B16F10 cells (1 × 10^4^ cells) in 100 μL of serum-free 1640 medium were added to all wells and incubated for 2 h at 37 °C. Afterwards, the unbound cells were removed by washing the wells two times with serum-free 1640 medium. Adherent cells were fixed with 4% (*w*/*v*) paraformaldehyde for 1 h at RT, rinsed twice in PBS, and stained with 1% (*w*/*v*) crystal violet for 30 min at RT. Following two times of washing, the stained cells were counted. For the adhesion inhibiting assay, cell suspensions were incubated with the anti-α5β1 monoclonal antibody (Bioss bs-1310R; 1:500) or anti-αvβ3 monoclonal antibody (Sino Biological CT014-MM01, Beijing, China; 1:500) for 1 h at RT. Then, the assay was performed in the same way as the adhesion assay.

### 2.14. Western Blot Assay

The B16F10 were seeded in 6-well plates (5 × 10^5^ cells/well) and treated with 0.25 μM HSA-HM-3 or 0.25 μM HM-3-HSA for 48 h. The protein samples were extracted and separated on 12% SDS-PAGE and transferred onto the PVDF membrane. Then, the non-specific site was blocked with 1% BSA for 2 h at RT and the membrane was incubated with diluted specific primary antibodies (Immunoway, Plano, TX, USA: rabbit polyclonal anti-FAK (YT1659), rabbit anti-phospho-FAK (YP0629); Affinity Biosciences, Cincinnati, OH, USA: anti-Src (AF6161); Thermo Fisher, Waltham, MA, USA: mouse monoclonal anti-phospho-Src (14-9034-82)) at 4 °C overnight. The membrane was further incubated for 1 h with a peroxidase-conjugated secondary antibody at RT. The immunoreactive proteins were detected by using an enhanced chemiluminescence (ECL) western blotting detection kit (New cell & Molecular biotech Co., Zhousu, China). GAPDH was used as the internal reference. The intensity of the bands was quantified using Image Lab software (Bio-Rad, Redmond, WA, USA).

### 2.15. Tumor Implantation and Treatment

B16F10 cells were collected at 1000 rpm for 5 min and resuspended in PBS, cell concentration was adjusted to 5 × 10^5^, and inoculated subcutaneously on the mid-left side of C57BL/6 female mice. After the average tumor volume grew to 60–80 mm^3^, the tumor-bearing mice were randomly divided into four groups (*n* = 6): (a) control group (treated with 0.1 mL PBS per 3 days); (b) HM-3 group (treated with HM-3 at a dose of 0.2 μmol/kg in 0.1 mL of PBS per 3 days); (c) HSA-HM-3 group (treated with HSA-HM-3 at a dose of 0.2 μmol/kg in 0.1 mL of PBS per 3 days); and (d) HM-3-HSA group (treated with HM-3-HSA at a dose of 0.2 μmol/kg in 0.1 mL of PBS per 3 days). PBS, HM-3, HSA-HM-3, or HM-3-HSA was applied to mice by s.c. injection. The tumor volumes were calculated using the formula: V = L × W^2^ × 0.5 (V: tumor volume, L: length, and W: width).

### 2.16. Immunohistochemistry

Mice were euthanized after 12 days post-treatment. The immunohistochemistry assay was done as described previously [20], with some modifications. Tumor tissues were stripped and fixed with 4% paraformaldehyde. Tumor tissues were embedded in paraffin and cut into sections for immunohistochemical analysis for Ki-67 (mouse anti- Ki-67 polyclonal antibody, Abcam Co., Cambridge, UK) to detect the proliferation of the tumor. In this section, the Ki-67 positive cells rate was evaluated in photomicrographs of tumor tissues (*n* = 3) with 200 × magnification in four fields of the tumor.

### 2.17. Analytic Methods

Numerical data were expressed as mean ± standard deviation (SD). For multiple comparisons, one-way ANOVA was performed using SPSS 23.0 software (SPSS Inc., Chicago, IL, USA). The significance level was defined as *p* < 0.05.

## 3. Results

### 3.1. Expression and WB Identification of the Fusion Proteins

**Figure 1:**
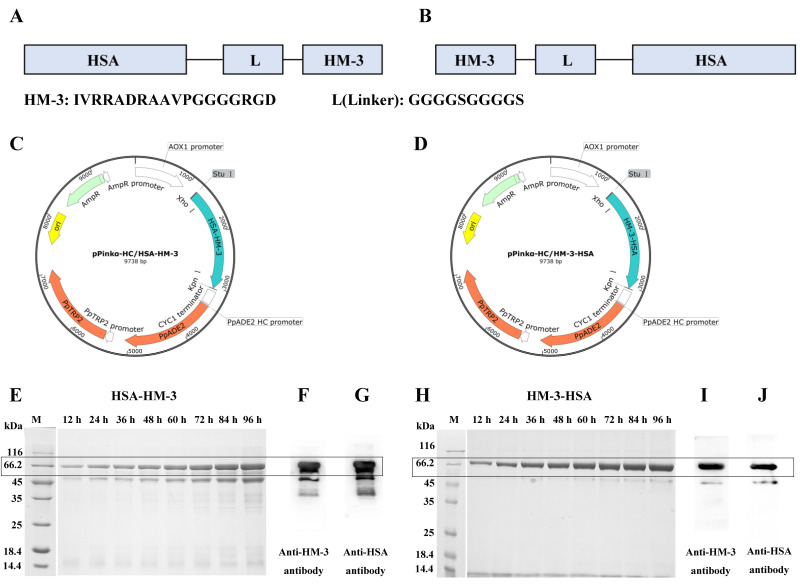
The expression of HM-3/HSA fusion proteins. (**A**,**C**) Design and construction of the pPinkα-HC-*HSA-HM-3* plasmid. (**B**,**D**) Design and construction of the pPinkα-HC-*HM-3*-*HSA* plasmid. (**E**) SDS-PAGE analysis of HSA-HM-3 expressed in yeast. (**F**) Western blot analysis of HSA-HM-3 by the monoclonal antibody against HM-3. (**G**) Western blot analysis of HSA-HM-3 by the polyclonal antibody against HSA. (**H**) SDS-PAGE analysis of HM-3-HSA expressed in yeast. (**I**) Western blot analysis of HM-3-HSA by the monoclonal antibody against HM-3. (**J**) Western blot analysis of HM-3-HSA by the polyclonal antibody against HSA.

*HSA* was genetically fused to the N-terminus (Figure 1A) or C-terminus (Figure 1B) of HM-3 through the GGGGSGGGGS linker. HSA-HM-3 or HM-3-HSA was then cloned into the pPinkα-HC vector (Figure 1C,D) and followed by the expression in *Pichiapink* strain. The obtained fusion proteins were analyzed by reducing SDS-PAGE. In Figure 1E, 1H showed that two fusion proteins were successfully expressed and secreted outside the cell. The western blot confirmed that two fusion proteins contained the epitopes of HM-3 (Figure 1F,I) and HSA (Figure 1G,J). The molecular weight of two fusion proteins were approximately 68 kDa, which corresponds to the theoretical molecular weight of HSA-HM-3 and HM-3-HSA. The production of HSA-HM-3 and HM-3-HSA were 205 mg/L and 218 mg/L, respectively (Table 1). However, serious degradation was observed and two distinct overlapping bands appeared in the major desired position of approximately 68 kDa on reducing SDS-PAGE (Figure 1E) and the western blot analysis (Figure 1F,G) of HSA-HM-3.

### 3.2. Purification and HPLC Analysis of the Fusion Proteins

**Figure 2:**
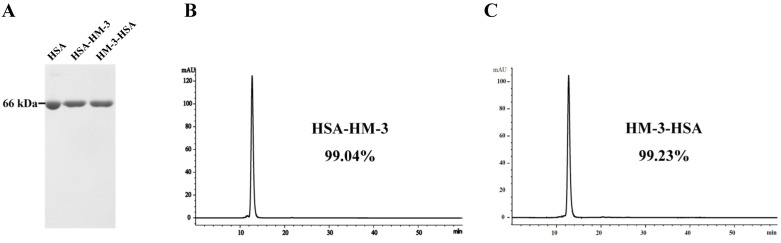
The purification and identification of HM-3/HSA. (**A**) Non-reducing SDS-PAGE analysis of purified HM-3/HSA. (**B**,**C**) Purified HM-3/HSA was analyzed in HPLC. Column: Protein-Pak^TM^ 125A, 7.8 × 300 mm column; PN: WAT084601. The mobile phase involved: 50 mM PB, 1.0 M NaCl solution; flow: 0.5 mL/min; temperature: 25 °C; wavelength: 280 nm; and injection volume: 10 μL.

The expressed supernatant that contained the fusion proteins was purified by DEAE Sepharose 6 Fast Flow chromatography and Phenyl Sepharose Fast Flow chromatography. As shown in Figure 2A, non-reducing SDS-PAGE analysis of the fusion proteins revealed a single band corresponding to the expected molecular weight. HPLC analysis confirmed that the purity of HSA-HM-3 and HM-3-HSA was more than 99% (Figure 2B,C). The purified HM-3/HSA fusion proteins were all soluble and the final concentrations were 618 mg/L (HSA-HM-3) and 651 mg/L (HM-3-HSA), respectively (Table 1).

### 3.3. Stability of Fusion Proteins

**Figure 3:**
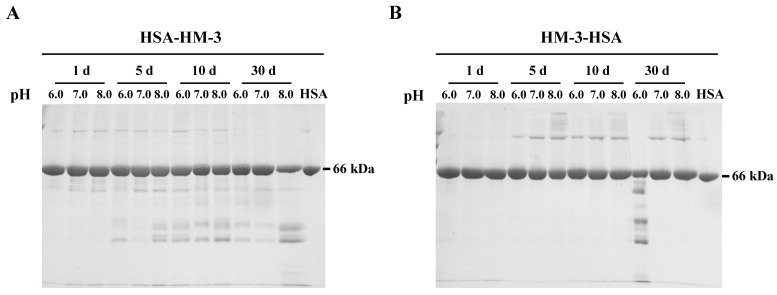
Stability of HSA-HM-3 (**A**) and HM-3-HSA (**B**) at different pH after being stored at 37 °C for 1, 5, 10, and 30 days.

As HSA and HM-3 exhibited maximal stability at neutral pH [21,22], only a narrow range of pH (pH 6.0, 7.0, and 8.0) were scouted for HM-3/HSA during stability tests. As shown in Figure 3A,B, HSA-HM-3 and HM-3-HSA exhibited maximal stability at pH 7.0 phosphate buffer. HSA-HM-3 was more susceptible to hydrolysis than HM-3-HSA at pH 7.0 and 8.0 at 30 days.

### 3.4. Circular Dichroism Spectral Analysis

**Figure 4:**
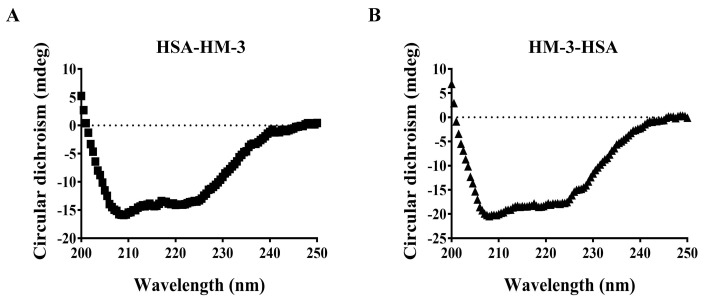
Far UV spectroscopy of HSA-HM-3 (**A**) and HM-3-HSA (**B**). The secondary structure of fusion proteins was analyzed by far-UV CD spectroscopy (200–250 nm). Bandwidth: 1 nm; scanning speed: 200 nm/min; D.I.T.: 0.5 sec; data pitch: 0.5 nm; and temperature: 25 °C.

Circular dichroism (CD) spectroscopy was used to obtain structure information of HSA-HM-3 and HM-3-HSA. The obtained CD data was deconvoluted to give the predicted secondary structure as shown in (Figure 4A,B).

The percent composition of the four main secondary structure components calculated by CDNN deconvolution analysis of the obtained spectra (Table 2) consists of 46% alpha helix, 10% parallel, 15% beta turn, and 28% random coil for HSA-HM-3, and 56% alpha helix, 8% parallel, 12% beta turn, and 22% random coil for HM-3-HSA, respectively. Compared with HSA-HM-3, the percentage of alpha helix of HM-3-HSA increased, while the random coil reduced.

### 3.5. Pharmacokinetic Analysis

The concentration of HSA-HM-3 and HM-3-HSA were detected by ELISA at 5 min, 30 min, 1 h, 2 h, 3 h, 6 h, 12 h, 24 h, and 48 h after single tail vein injection. The individual pharmacokinetic parameters of fusion proteins in mice were summarized in Table 3. The time-drug concentration equation was acquired, namely y = 7.228 − 7.085/(1 + x/49.68) ^ 0.950, R^2^ = 0.99996. The non-compartmental analysis of serum data suggested that the biological half-life of HSA-HM-3 and HM-3-HSA in circulation were 14 h and 17 h respectively. Meanwhile, serum concentration of HSA-HM-3 reached a maximum value at about 6 h, whereas HM-3-HSA was at about 7 h. The apparent clearance of HSA-HM-3 and HM-3-HSA were 140 L/h/kg and 156 L/h/kg, respectively, and the volumes of distribution were 3032 L/kg and 3923 L/kg, respectively.

### 3.6. HM-3/HSA Inhibited B16F10 Cells’ Viability

**Figure 5:**
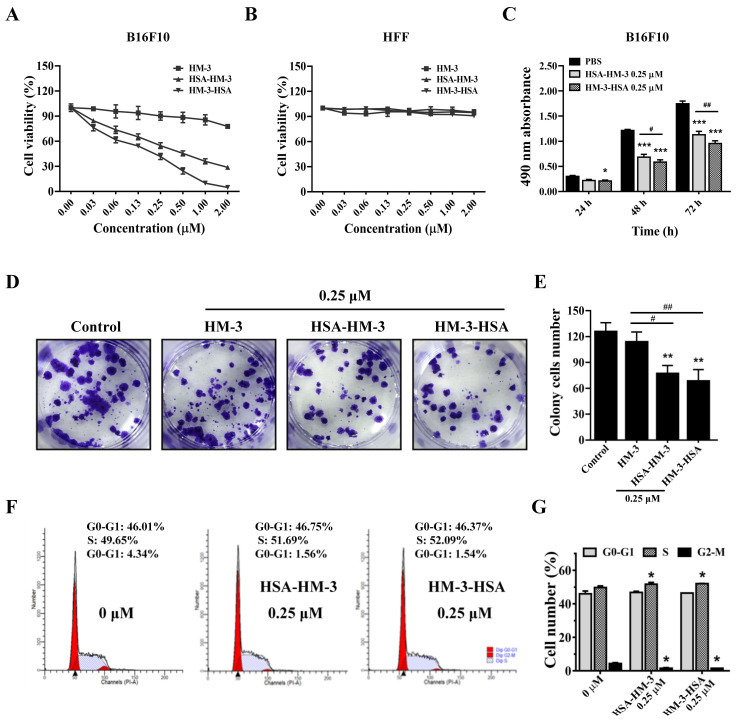
The effect of HM-3/HSA on B16F10 cells’ viability. (**A**,**B**) HM-3/HSA inhibited B16F10 and HFF cells’ viability in a dose-dependent manner. Cells were incubated with various doses of HSA-HM-3 or HM-3-HSA for 48 h, followed by cell viability evaluation using the MTT assay. (**C**) HM-3/HSA inhibited B16F10 cells’ viability within 72 h. Cells were incubated with HSA-HM-3 (0.25 μM) and HM-3-HSA (0.25 μM) for 24, 48, and 72 h, followed by cell viability evaluation using the MTT assay. (**D**,**E**) HM-3/HSA inhibited B16F10 cells’ colony formation. Cell proliferation was measured by the colony formation assay in B16F10 cells treated with the indicated concentrations of HM-3/HSA for 7 days. (**F**,**G**) HM-3/HSA induced S-phase cell cycle arrest in B16F10 cells. The analysis of cell cycle arrest was done by the PI staining assay after 48 h of incubation with HM-3/HSA and cell-cycle distribution was determined by flow cytometry. Control: cells treated with the PBS vehicle only. Data shown are means ± standard deviation from three independent experiments. * *p* < 0.05, ** *p* < 0.01, and *** *p* < 0.001 vs. control; ^#^ *p* < 0.05 and ^##^ *p* < 0.01.

To assess the effect of HM-3/HSA on the viability of B16F10 cells, the MTT assay was carried out. As illustrated in Figure 5A, it was observed that the viability of B16F10 cells was markedly inhibited after exposure of HM-3/HSA in a dose-dependent manner; however, no significant inhibitory effect was observed in HFF cells (Figure 5B). The IC_50_ values of HSA-HM-3 and HM-3-HSA were calculated as 0.38 ± 0.14 μM and 0.25 ± 0.08 μM in B16F10 cells, respectively. In comparison, the IC_50_ values of HM-3 was 30.07 ± 9.19 μM. In a time-dependent manner, results demonstrated that B16F10 cells’ viability was inhibited within 72 h of HSA-HM-3 or HM-3-HSA treatment (Figure 5C).

In addition, we evaluated the effect of HM-3/HSA on the colony formation, which is a characteristic of tumor cells and is closely related to carcinogenesis [23]. As shown in Figure 5D,E, the colony forming ability of B16F10 cells was significantly decreased by HSA-HM-3 and HM-3-HSA relative to the control. The inhibition rates were 38% and 45% by HSA-HM-3 and HM-3-HSA, respectively, whereas the inhibition rate was 9% by that of HM-3.

After treating with HM-3/HSA for 48 h, the B16F10 cells were then stained with PI. As shown in Figure 5F,G, the percentage of B16F10 cells in the S phase significantly increased when cells were treated with HM-3/HSA, while the percentage of cells in the G2-M phase significantly decreased. These results indicated that the transformation of cells from the S to G2-M phase was blocked after exposure of HM-3/HSA, thereby inhibiting cell proliferation.

Overall, HM-3/HSA showed the higher inhibitory effect on B16F10 cells’ viability than HM-3 in vitro and when HSA was fused at the C-terminus of HM-3, the inhibitory effect was better than when fused at the N-terminus.

### 3.7. HM-3/HSA Inhibited B16F10 Cells’ Migration and Invasion

**Figure 6:**
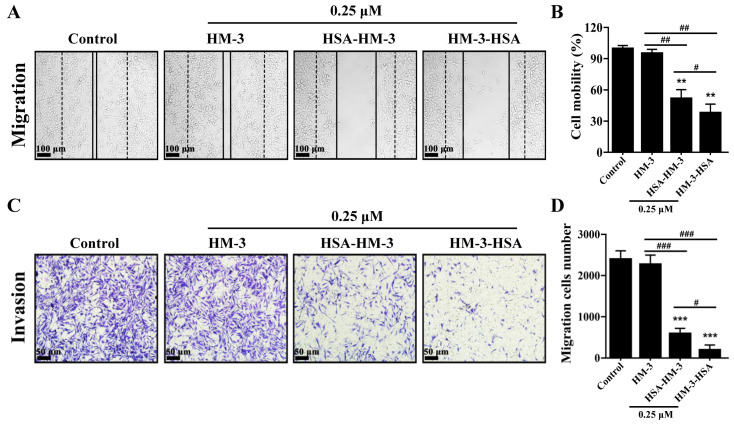
The effect of HM-3/HSA on B16F10 cells’ migration and invasion. (**A**,**B**) HM-3/HSA inhibited B16F10 cells’ migration. Cell migration of B16F10 cells was assessed by the wound-scratch assay. The dotted line indicates the baseline immediately at the wound scratch, whereas the solid line indicates the migration at 48 h after HM-3, HSA-HM-3, or HM-3-HSA treatment. Scale bar = 100 μm. (**C**,**D**) HM-3/HSA inhibited B16F10 cells’ invasion. Cell invasion of B16F10 cells was assessed by the trans-well assay after HM-3, HSA-HM-3, or HM-3-HSA treatment for 48 h. Scale bar = 50 μm. Control: cells treated with the PBS vehicle only. Data shown are means ± standard deviation from three independent experiments. ** *p* < 0.01 and *** *p* < 0.001 vs. control; ^#^ *p* < 0.05, ^##^ *p* < 0.01, and ^###^ *p* < 0.001.

To assess the effect of HM-3/HSA on metastatic activity, we investigated the migration and invasion of B16F10 cells using the wound scratch and trans-well system, respectively. Figure 6A,B shows that HM-3/HSA suppressed the closure rate of the scratch at 48 h treatment compared with control. Among them, the cell mobility rates were 52% and 38% of control by HSA-HM-3 and HM-3-HSA, respectively, but the cell mobility rate was 95% by HM-3 at the same concentration.

Moreover, two fusion proteins apparently decreased the invasion of B16F10 cells in the trans-well chamber assay (Figure 6C,D). The inhibition rates of invasion were 75% and 89% by HSA-HM-3 and HM-3-HSA, respectively. However, the inhibition rate was only 5% by HM-3 at the same concentration. 

These findings suggested that HM-3/HSA showed the higher inhibitory effect on B16F10 cells’ migration and invasion than that of HM-3 and when HSA was fused at the C-terminus of HM-3, the anti-migration and anti-invasion activity was better than when fused at the N-terminus.

### 3.8. HM-3/HSA Bind to Integrin and Inhibited Integrin Signaling Pathways

**Figure 7:**
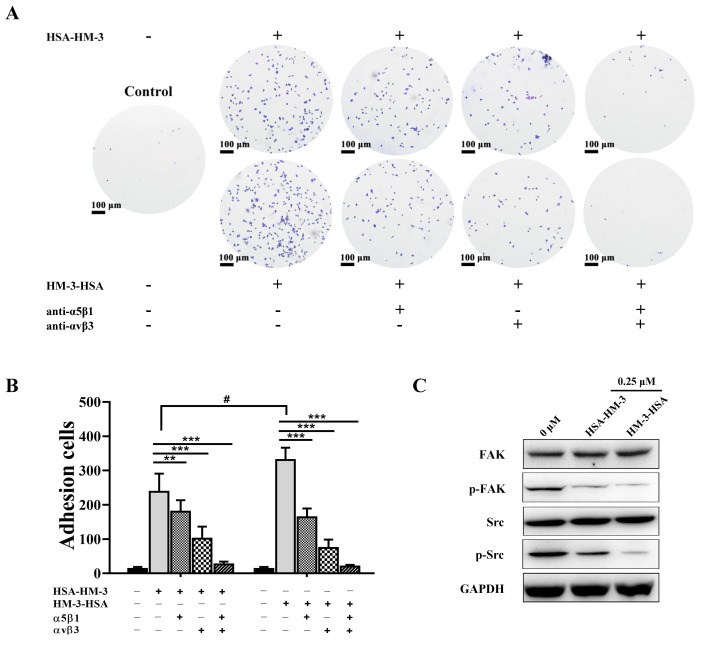
HM-3/HSA inhibited FAK/Src signaling pathways by binding with integrin αvβ3 and α5β1. (**A**,**B**) The integrin monoclonal antibody-blocking assay was performed to verify the interaction between HM-3/HSA and both integrin α5β1 and αvβ3. The high binding rate of HM-3/HSA increased with B16F10 cells through binding with integrin αvβ3 and α5β1 of the cell surface, but the adhesion number was significantly decreased when B16F10 cells were blocked with the integrin αvβ3 or α5β1 antibody. Control: cells treated with the PBS vehicle only. Scale bar = 100 μm. Data shown are means ± standard deviation from three independent experiments. ** *p* < 0.01, *** *p* < 0.001, and ^#^ *p* < 0.05. (**C**) HM-3-HSA affected the FAK/Src signaling pathway in B16F10 cells. The B16F10 cells were treated with 0.25 μM HSA-HM-3 or 0.25 μM HM-3-HSA for 48 h. GAPDH was used as the internal reference. Data shown are means ± standard deviation from two independent experiments.

B16F10 cells are metastatic cancer cells with high expression levels of integrin α5β1 [24] and αvβ3 [25]. The integrin antibody-blocking assay was performed to verify the interaction between HM-3/HSA and both integrin α5β1 and αvβ3. As shown in Figure 7A,B, we found that B16F10 cells exhibited a significant adhesion to the plates coated with HM-3/HSA, indicating HM-3/HSA has a high binding rate with B16F10 cells. However, the adhesion number was significantly decreased when B16F10 cells were blocked with the integrin αvβ3 or α5β1 antibody. The adhesion number was almost completely inhibited when B16F10 cells were blocked with the integrin αvβ3 and α5β1 antibody, suggesting that the binding of HM-3/HSA with B16F10 cells was associated with integrin αvβ3 and α5β1 expression. Taken together, our findings indicated that HM-3/HSA was properly folded and thus showed a high binding rate with B16F10 cells through integrin αvβ3 and α5β1 of the cell surface. In addition, when HSA was fused at the C-terminus of HM-3, the adhesion cell number without the integrin antibody was higher than when fused at the N-terminus.

The activation of integrin leads to the activation of downstream signaling pathways. FAK is one of the major substrates of integrin and can further phosphorylate and activate downstream signaling molecules Src [26]. It has been reported that the FAK/Src signaling pathway is crucial for the proliferation, migration, and invasion of tumor cells [27]. As shown in Figure 7C, western blotting analysis demonstrated that the cells treated with HM-3/HSA had lower levels of phospho-FAK and phospho-Src, while the total protein FAK and Src levels were unaffected following HM-3/HSA treatment.

These results suggested that HM-3/HSA exerted the bioactivities by binding with integrin αvβ3 and α5β1 on the surface of B16F10 cells and further blocked the integrin-mediated FAK/Src signal pathway.

### 3.9. HM-3/HSA Inhibited the Growth of the B16F10 Tumor

**Figure 8:**
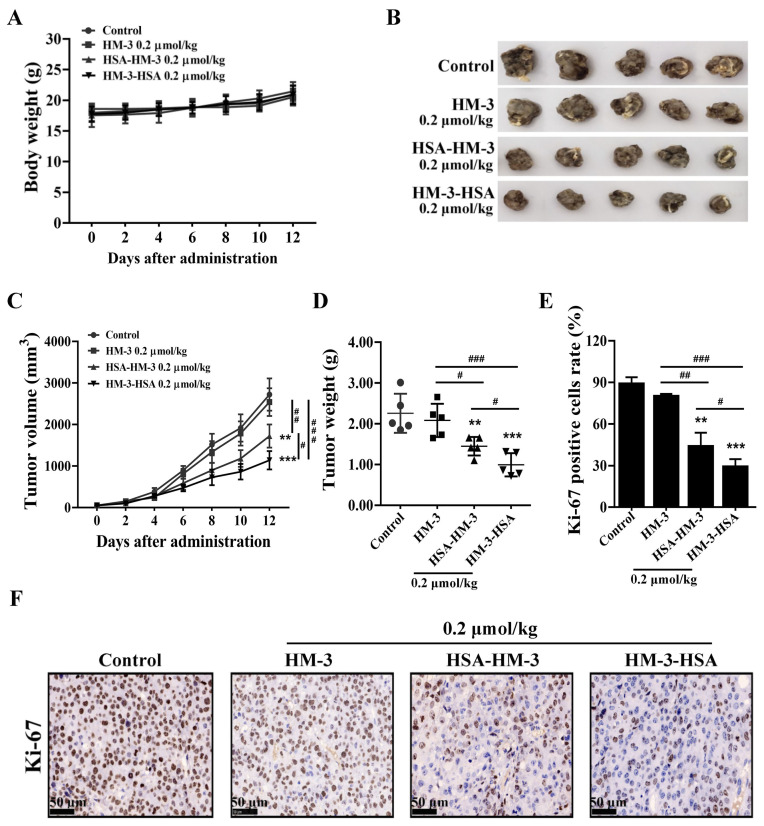
The effect of HM-3/HSA on the growth of the B16F10 tumor. (**A**) The body weight of mice. (**B**) Tumor picture. (**C**) HM-3/HSA inhibited the B16F10 tumor volume (*n* = 6 mice/group). The volume was measured at 2 d, 4 d, 6 d, 8 d,10 d, and 12 d after administration. (**D**) HM-3/HSA inhibited the B16F10 tumor weight. Tumor weight in different treatment groups was measured at the end of treatment. (**E**) The Ki-67 expression level of B16F10 melanoma tissues was counted and presented in the histogram. (**F**) Ki-67 immunohistochemistry pictures of tumor tissues. Control: mice treated with the PBS vehicle only. Scale bar = 50 μm. ** *p* < 0.01, and *** *p* < 0.001 vs. control; ^#^ *p* < 0.05, ^##^ *p* < 0.01, and ^###^ *p* < 0.001.

Previous study indicated that HM-3-HSA at 0.2 μmol/kg could present better antitumor activity than other concentrations in the B16F10 tumor model (WO2020238924 A1). Therefore, 0.2 μmol/kg HM-3/HSA was chosen to be used in the experiments and 0.2 μmol/kg HM-3 was used as a control. The results suggested that the three treatment groups had no effect on the mice body weight (Figure 8A). Compared with the control, HM-3/HSA exerted a certain tumor inhibitory effect after 12 days of administration **(**Figure 8B,C). In terms of tumor weight, the inhibition rates were 36% and 56% of control by HSA-HM-3 and HM-3-HSA, whereas HM-3 only caused 8% inhibition (Figure 8D).

Tumor tissues were collected and fixed. Frozen tumor specimens were cut into sections for Ki-67 immunohistochemical staining to verify the proliferation level of the tumor. As illustrated in Figure 8E,F, Ki-67 positive cell rates were 37% and 29% by HSA-HM-3 and HM-3-HSA, whereas HM-3 was 79%.

Collectively, these findings revealed that the antitumor efficacy of HM-3/HSA was higher than that of HM-3 in vivo and when HSA was fused at the C-terminus of HM-3, the anti-tumor activity was better than when fused at the N-terminus.

## 4. Discussion

HM-3 is a promising antitumor polypeptide and has recently entered clinical trials in China to treat solid tumors (CTR20200847). However, its short circulating half-life makes frequent administration necessary, which limits its clinical uses [6]. Two protein modification strategies have been employed to improve its in vivo pharmacokinetics: PEGylated HM-3 and Fc-fused HM-3. Both PEGylated HM-3 and Fc-fused HM-3 have high biological activities and good pharmacokinetic profiles [5,6,28]. Compared with Fc and PEG, HSA has the better biochemical and physiological properties for drug delivery, such as long blood circulation, a wide distribution, a weak immunogenicity, and tumor targeting.

Motivated by the promising characteristics of HM-3 peptide, HSA was genetically fused to the N or C-terminal of HM-3, named HSA-HM-3 and HM-3-HSA. Both HSA-HM-3 and HM-3-HSA displayed a prolonged half-life of over 14 h in mice, which is about 30-folds higher than that of HM-3 (27 min) [2]. The favorable blood circulation profile of HM-3/HSA will improve the clinical applicability, which would allow for less injection frequency and maintain a more constant circulating level. Moreover, the bioactivities of HM-3/HSA was higher than HM-3 at the same concentration in vitro. Previous studies showed that when HSA was fused to hLF, it exerted better bioactivity in cancer cells than the unmodified hLF [14]. Consistent with in vitro results, HM-3/HSA has a better antitumor activity than HM-3 in vivo, which may be related to its prolonged half-life and tumor targeting [16]. Tian et al. [29] and Huang et al. [30] reported the same phenomenon on HSA-IFNα1 and HSA-IFNα2b fusion proteins, respectively. Further studies are needed to elucidate the mechanisms underlying the enhanced antitumor effects of HSA-fused HM-3. In summary, favorable pharmacokinetic characteristics and potent bioactivity make HM-3/HSA a promising candidate HM-3 agent for treatment of melanoma.

Although HSA has been widely exploited as a fusion partner to extend the in vivo half-life of therapeutic proteins and both the N-terminal and C-terminal fusion proteins have been shown to improve the pharmacokinetics of therapeutic proteins [14,17,31,32,33], the effect of fusion orientation on the expression level, stability, and activity of the HSA fusion protein have not been extensively studied. In our study, no significant production difference occurred between HSA-HM-3 and HM-3-HSA, indicating that the fusion orientation of HSA does not contribute to the changes in the yield of the fusion protein. Although similar expression levels of two fusion proteins were found, different characteristics were observed. The purified HSA-HM-3 was more prone to hydrolysis than HM-3-HSA as judged by stability measurements (Figure 3). Although the aggregates were observed both for HSA-HM-3 and HM-3-HSA, we have not found the compromise of aggregates-to-fusion protein activity fortunately (Figure S1). In addition, the result of far-UV CD indicated that the percentage of the alpha helix of HM-3-HSA was more than HSA-HM-3, while random coil was decreased. The decrease in the α-helix structure and increase in the random coil could cause a partial protein unfolding [34]. Thus, the differences between the HSA-HM-3 and HM-3-HSA secondary structure may be closely correlated to their different stability. Subsequent pharmacokinetic studies also found that HM-3-HSA has a longer half-life (17 h) than HSA-HM-3 (14 h), Fc-fused HM-3 (15 h) [5], and PEGylated HM-3 (162 min) [6].

It has been reported that HM-3 could exert bioactivities by binding to integrin αvβ3 and αvβ5 of the cell surface [1,2]. Our results showed that two fusion proteins could also bind to B16F10 cells through integrin αvβ3 and α5β1 of the cell surface, and inhibit the phosphorylation of FAK and Src downstream signaling molecules of the integrin. This result indicated that the fusion of HSA does not change the targets of HM-3 and HM-3/HSA was properly folded. Moreover, we found that the binding rate of HM-3-HSA with B16F10 cells was higher than that for HSA-HM-3 (Figure 7A,B). Interestingly, both in vivo and in vitro bioactivity assays showed that HM-3-HSA could result in higher activity than HSA-HM-3, which may be related to its binding efficiency with B16F10 cells. Previous studies showed that some small molecules fused at the N-terminus of HSA exhibited higher activities than that at the C-terminus, such as hirudin variant 3 (HV3) [35], somatostatin-14 [32], somatostatin-28 [31], human tumor necrosis factor receptors (shTNFRI) [36], and human lactoferrin (hLF) [14]. For HM-3, one possible explanation is that HSA fusing at the N-terminus of HM-3 might cause a larger steric hindrance effect or may influence post-translational folding and receptor binding, while fusion at the C-terminus of HM-3 seems to reduce the influence to some extent. After subsequent work, we will discuss the reason for the effects of the fusion orientation on the bioactivity of HSA fusion proteins to provide a theoretical basis for the construction of HSA fusion proteins.

In addition, HM-3/HSA facilitated the growth inhibition effects in cancer cells but no significant influence was observed for normal cells. Interestingly, HM-3/HSA could inhibit the viability of B16F10 cells by arresting the cell cycle instead of inducing cell apoptosis (Figure S2). These results indicated a high safety profile for HM-3/HSA.

Overall, we have developed two novel fusion proteins that consist of HSA genetically fused at the N or C-terminus of HM-3, termed HSA-HM-3 and HM-3-HSA. Two fusion proteins were successfully expressed in *Pichia pastoris*. HM-3/HSA showed a more improved pharmacokinetic profile than HM-3. HM-3-HSA showed higher antitumor activity than HSA-HM-3 in vitro and in vivo. This is a new avenue towards improving the therapeutic property of HM-3 based on genetic fusion technology.

## Figures and Tables

**Table 1:** Purification process and recovery of HM-3/HSA.

Purification Step	HSA-HM-3			HM-3-HSA		
	Volume (mL)	Concentration (mg/L)	Recovery (%)	Volume (mL)	Concentration (mg/L)	Recovery (%)
Culture supernatant	1000	205.5	100	1000	218.6	100
DEAE Sepharose 6 Fast Flow	355	492.0	85.0	391	481.9	86.2
Phenyl Sepharose Fast Flow	151	618.9	53.5	149	651.3	51.5

**Table 2:** Comparison of secondary structures of HSA-HM-3 and HM-3-HSA *.

Parameter	HSA-HM-3	HM-3-HSA
Alpha helix	46.4	56.3
Parallel	10.1	8.1
Beta-turn	15.2	12.8
Random coil	28.3	22.8

* The relative amounts of different secondary structures in HSA-HM-3 and HM-3-HSA were calculated from the spectral data shown in Figure 4 by CDNN deconvolution analysis. The average value of each secondary structure component following the four sets of measurements is shown as percentages.

**Table 3:** Pharmacokinetic parameters of HM-3/HSA after a single administration in KM mice *.

Parameter	HSA-HM-3	HM-3-HSA
t_1/2_ (h)	14.99	17.36
CLz (L/h/kg)	140.23	156.62
AUC(0−t) (μg/L *h)	127.99	110.43
AUC (0−∞) (μg/L *h)	142.62	127.70
Vz (L/kg)	3032.95	3923.60
C_max_ (μg/L)	5.99	7.09

* t_1/2_ = the half-life in serum. Abbreviations: CLz = apparent clearance following tail vein administration; AUC (0−t) = area under the curve until the last measurable concentration; AUC (0−∞) = AUC from time zero to infinity; Vz = apparent volume of distribution; and C_max_ = maximum concentration of HM-3/HSA.

## Data Availability

The data that support the findings of this study are available from the corresponding author upon reasonable request.

## Supplementary Materials

The following are available online at https://www.mdpi.com/article/10.3390/biomedicines9091084/s1, Figure S1: The anti-proliferation activity of HM-3-HSA at pH 7.0 after being stored at 37 °C for 1 and 5 days; Figure S2: HM-3/HSA had no effect on cell apoptosis; and Table S1: List of primers used in the construction of pPinkα-HC/HSA-HM-3 and pPinkα-HC/HM-3-HSA.

## Abbreviations

DMSO	dimethyl sulfoxide
FBS	fetal bovine serum
PBS	phosphate buffer saline
HSA	human serum albumin
MTT	3-(4,5-dimethylthiazol-2-yl)-2,5-diphenyltetrazolium bromide
PCR	polymerase chain reaction
CD	circular dichroism
