# Supplementary material for: Albumin Fusion at the N-Terminus or C-Terminus of HM-3 Leads to Improved Pharmacokinetics and Bioactivities
Source: Biomedicines. 2021 Aug 25;9(9):1084. doi: 10.3390/biomedicines9091084 (PMC8472738; doi:10.3390/biomedicines9091084)
Supplement: Supplementary file 1 [file biomedicines-09-01084-s001.zip › biomedicines-1354770-supplementary.pdf]

Supplementary materials

Table S1

Table S1. List of primers used in construction of pPinkα-HC/HSA-HM-3 and pPinkα-HC/HM-3-HSA.

Primer	Sequence (5′-3′)
P_{HSA}	GATGCTCATAAGTCTGAAGTTG
R_{HSA}	TTACAAACCCAAAGCAGCTTGAGAAGC
P_1	GAAGGCCTGATGCTCATAAGTCTGAAGTTGC
R_1	TAGAACCACCACCACCAGAACCACCACCACCCAAACCCAAAGCAGCTTGAGA
R_2	CCTGGAACAGCAGCTCTATCAGCTCTTCTAACAATAGAACCACCACCACCAGAAC
R_3	CGGTACCTTAGTCACCTCTACCACCACCACCTGGAACAGCAGCTCTATC
P_2	GGTGGTGGTGGTTCTGGTGGTGGTGGTTCTGATGCTCATAAGTCTGAAGTTGC
P_3	GCTGCTGTTCCAGGTGGTGGTGGTAGAGGTGACGGTGGTGGTGGTTCTGGTGGT
P_4	GAAGGCCTATTGTTAGAAGAGCTGATAGAGCTGCTGTTCCAGGTGGTGGT
R_4	CGGTACCTTACAA ACCCAAAGCAGCTTGAGAAGC

Figure S1

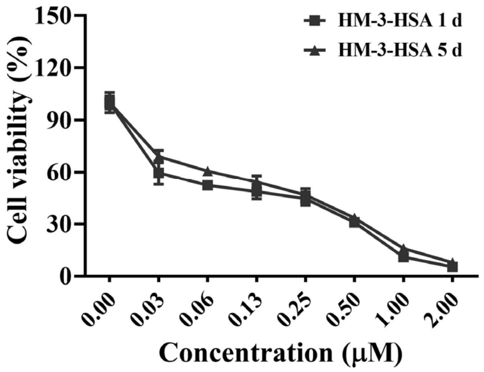

Figure S1. The anti-proliferation activity of HM-3-HSA at pH 7.0 after stored at 37°C for 1 and 5 days. Cells were incubated with various doses of HM-3-HSA for 48 h followed by cell viability evaluation using MTT assay. Data shown are means ± standard deviation from three independent experiments.

Figure S2

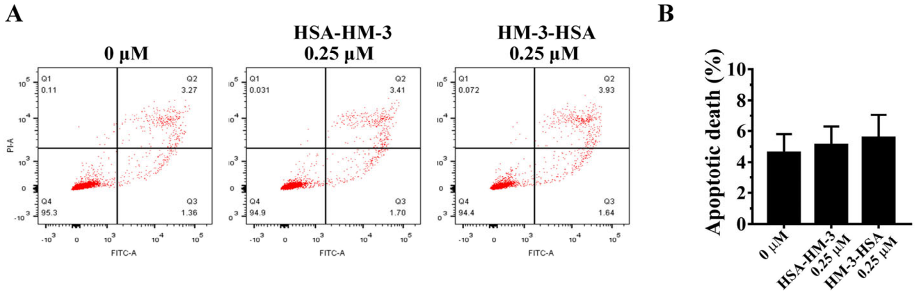

Figure S2. HM-3/HSA had no effect on cell apoptosis. (A, B) HM-3/HSA could not induce cell apoptosis in B16F10 cells. Cells were treated with the indicated concentration of HM-3/HSA for 48 h, and the level of apoptosis was determined using an An-nexin-V-FITC/PI double staining assay. Control: Cells treated with PBS vehicle only. Data shown are means ± standard deviation from three independent experiments.
